# Combined Cardiac Impairments in End-Stage Renal Disease

**DOI:** 10.1007/s00246-012-0422-5

**Published:** 2012-07-13

**Authors:** Małgorzata Sobieszczańska, Krystyna Laszki-Szcząchor

**Affiliations:** Wroclaw Medical University, Wroclaw, Poland

To the Editor,

We were impresses by the article published in *Pediatric Cardiology* by Kobayashi et al. [1], The Impact of Change in Volume and Left-Ventricular Hypertrophy on Left-Ventricular Mechanical Dyssynchrony in Children With End-Stage Renal Disease. The study provides an important contribution to the scarce data on functional/organic cardiac impairments in children with end-stage renal disease (ESRD).

Kobayashi et al. detected left-ventricular dyssynchrony (LVD) using real-time three-dimensional echocardiography in children with ESRD undergoing renal replacement therapy (peritoneal dialysis or hemodialysis). The authors stated that dyssynchrony was significantly greater in ESRD children, especially those on hemodialysis therapy, compared with controls and improved after hemodialysis. The authors concluded that ESRD children presented volume load and left-ventricular hypertrophy (LVH), which were probably associated with increased LVD. They assumed that cardiac dyssynchrony could be an independent predictor of serious cardiac events in patients with ESRD [1].

Regarding cardiac impairments in such patients, we would like to draw attention to our findings [2, 3] showing intraventricular conduction disturbances in ESRD children in whom no abnormalities were observed on 12-lead electrocardiographic and echocardiographic examinations. In our study, a noninvasive multielectrode (87-lead) body surface potential mapping system (Fukuda Denshi Co Ltd, Tokyo, Japan) was applied to create isochrone maps to precisely reflect a sequence of ventricular activation time (i.e., VAT maps).

In our ESRD children treated conservatively [2] or with renal replacement [3], abnormal maps with delayed VAT values were noted, indicating some perturbations within the intraventricular conduction pathway. Patients on hemodialysis for <1 year demonstrated VAT maps typical for left bundle branch block (LBBB), which was partially alleviated after renal transplantation, becoming left anterior fascicle block. In children on hemodialysis for >1 year, VAT maps showed more serious disturbances in the form of complete LBBB that was normalized to incomplete LBBB due to kidney transplantation.

According to Kobayashi et al. [1], disorders of electrical activation, consequently leading to LVD, can occur in patients with ESRD. Multifactoral damage of the ventricular myocardium probably causes spatiotemporal heterogeneity and therefore LVD, which is additionally impaired by LVH. It is supposed that LVH can be a response of the myocardium to numerous “cross-talking networks” appearing both as the result of ESRD and during renal-replacement therapy.

Our findings [2, 3] suggest that disturbances of the intraventricular conduction system observed in children with ESRD can be a significant component of LVD, which inevitably leads to systolic left-ventricular dysfunction.

## References

[1] Kobayashi D, Patel SR, Mattoo TK, Valentini RP, Aggarwal S (2012) The impact of change in volume and left-ventricular hypertrophy on left-ventricular mechanical dyssynchrony in children with end-stage renal disease. Pediatr Cardiol. doi:10.1007/s00246-012-0266-z 10.1007/s00246-012-0266-z 22441563

[2] Laszki-Szcząchor K, Polak-Jonkisz D, Zwolińska D, Rusiecki L, Janocha A, Sobieszczańska M (2012). Heart ventricular activation in VAT difference maps from children with chronic kidney disease. Pediatr Nephrol.

[3] Polak-Jonkisz D, Laszki-Szcząchor K, Sobieszczańska M, Makulska I, Pilecki W, Rusiecki L (2011). Effect of kidney transplantation on heart conduction disturbances in children treated with chronic hemodialysis—a pilot study. Pediatr Transplant.

